# Non-Toxic Dimeric Peptides Derived from the Bothropstoxin-I Are Potent SARS-CoV-2 and Papain-like Protease Inhibitors

**DOI:** 10.3390/molecules26164896

**Published:** 2021-08-12

**Authors:** Marjorie C. L. C. Freire, Gabriela D. Noske, Natália V. Bitencourt, Paulo R. S. Sanches, Norival A. Santos-Filho, Victor O. Gawriljuk, Eduardo P. de Souza, Victor H. R. Nogueira, Mariana O. de Godoy, Aline M. Nakamura, Rafaela S. Fernandes, Andre S. Godoy, Maria A. Juliano, Bianca M. Peres, Cecília G. Barbosa, Carolina B. Moraes, Lucio H. G. Freitas-Junior, Eduardo M. Cilli, Rafael V. C. Guido, Glaucius Oliva

**Affiliations:** 1 São Carlos Institute of Physics, University of Sao Paulo, Avenida João Dagnone, 1100, São Carlos 13563-120, SP, Brazil; marjorie_freire_@hotmail.com (M.C.L.C.F.); gabriela.noske@usp.br (G.D.N.); victor.gawriljuk@gmail.com (V.O.G.); victor.nogueira@usp.br (V.H.R.N.); marianaort@gmail.com (M.O.d.G.); alinemnk@gmail.com (A.M.N.); rafaela.fernandes@usp.br (R.S.F.); andre_godoy@yahoo.com.br (A.S.G.); 2 Department of Biochemistry and Organic Chemistry, Institute of Chemistry, São Paulo State University (UNESP), Araraquara 14800-060, SP, Brazil; natalia.bitencourt@unesp.br (N.V.B.); paulo.sanches@unesp.br (P.R.S.S.); norival.santos-filho@unesp.br (N.A.S.-F.); eduardocilli@gmail.com (E.M.C.); 3 Department of Genetics and Evolution, Federal University of São Carlos, Rodovia Washington Luís km 235, São Carlos 13565-905, SP, Brazil; edupsouza96@gmail.com; 4 The Sao Paulo School of Medicine, Federal University of São Paulo, Rua Três de Maio, 100, São Paulo 04044-020, SP, Brazil; ma.juliano@unifesp.br; 5 Department of Microbiology, Institute of Biomedical Sciences, University of Sao Paulo, Av. Prof. Lineu Prestes, 1374, São Paulo 05508-900, SP, Brazil; biaperes04@gmail.com (B.M.P.); cecigomes.barbosa@gmail.com (C.G.B.); luciofreitasjunior@gmail.com (L.H.G.F.-J.); 6 Department of Pharmaceutical Sciences, Federal University of São Paulo, Rua São Nicolau, 210, Diadema 09913-030, SP, Brazil; cbmoraes@unifesp.br

**Keywords:** COVID-19, SARS-CoV-2, inhibitors, Papain-like protease, peptides

## Abstract

The COVID-19 outbreak has rapidly spread on a global scale, affecting the economy and public health systems throughout the world. In recent years, peptide-based therapeutics have been widely studied and developed to treat infectious diseases, including viral infections. Herein, the antiviral effects of the lysine linked dimer des-Cys^11^, Lys^12^,Lys^13^-(pBthTX-I)_2_K (**(pBthTX-I)_2_K)**) and derivatives against SARS-CoV-2 are reported. The lead peptide **(pBthTX-I)_2_K** and derivatives showed attractive inhibitory activities against SARS-CoV-2 (EC_50_ = 28–65 µM) and mostly low cytotoxic effect (CC_50_ > 100 µM). To shed light on the mechanism of action underlying the peptides’ antiviral activity, the Main Protease (M^pro^) and Papain-Like protease (PL^pro^) inhibitory activities of the peptides were assessed. The synthetic peptides showed PL^pro^ inhibition potencies (IC_50_s = 1.0–3.5 µM) and binding affinities (*K*_d_ = 0.9–7 µM) at the low micromolar range but poor inhibitory activity against M^pro^ (IC_50_ > 10 µM). The modeled binding mode of a representative peptide of the series indicated that the compound blocked the entry of the PL^pro^ substrate toward the protease catalytic cleft. Our findings indicated that non-toxic dimeric peptides derived from the Bothropstoxin-I have attractive cellular and enzymatic inhibitory activities, thereby suggesting that they are promising prototypes for the discovery and development of new drugs against SARS-CoV-2 infection.

## 1. Introduction

The Severe Acute Respiratory Syndrome virus 2 (SARS-CoV-2) is the causative agent of the Coronavirus disease 2019 (COVID-19), which was first reported in patients in Wuhan, China, in December 2019 [1]. Since then, the COVID-19 outbreak has rapidly spread on a global scale, affecting the economy and public health systems throughout the world [2]. As a result, in March 2020, the World Health Organization (WHO) declared COVID-19 as a pandemic disease, emphasizing the urgency in establishing strategies to contain the spread of SARS-CoV-2 [3]. Nearly two years have passed since the pandemic started and in August 2021, more than 202 million infection cases have been confirmed, with over 4.2 million deaths worldwide [4]. Although different vaccines have recently been approved, the vaccination of the world’s population will take time, which, together with the emergence of viral variants, highlights that the search for effective treatments remains a priority [5].

SARS-CoV-2 is a member of the *Coronaviridae* family and is part of the Beta-coronavirus genus, together with the SARS-CoV and MERS-CoV viruses that have been responsible for previous outbreaks in 2002 and 2012, respectively [6]. The viral genome is a single-stranded positive RNA of approximately 30,000 bases [7] that encodes four structural proteins (spike (S), nucleocapsid (N), membrane (M), and envelope protein (E)), forming the viral structure and also playing important roles in the recognition of cellular receptors, viral entry, and interaction with antibodies [8,9]. Additionally, 16 non-structural (NS) proteins (nsp1–nsp16) are encoded and participate in viral replication and pathogenesis [7,10].

The nsp3 gene encodes the cysteine protease papain-like (PL^pro^), which is responsible for viral polyprotein cleavage and processing [7]. PL^pro^ recognizes the tetrapeptide motifs LXGG located in the viral polyprotein pp1a and hydrolyzes the peptide bond on the carboxyl side of glycine at the P1 position, thereby releasing nsp1, nsp2, and nsp3 proteins [11]. Thus, this function is essential to generate the replicase complex and consequently enable the viral spread [12]. Moreover, PL^pro^ has shown deubiquinating activity that affects post-translational modifications on host proteins, contributing to the evasion of the host immune responses [12]. Due to its importance in the viral replication process, this enzyme is a promising molecular target in the discovery of new anti-SARS-CoV-2 agents [7,13].

Peptide-based therapeutics have been widely studied and developed to treat viral infections [14,15]. Moreover, the use of bioactive peptides presents advantages including high specificity, efficacy, broad spectrum activity, and safety. [15,16]. Peptide-based molecules have been investigated as inhibitors of viral proteins, such as Hemagglutinin A (HA) from Influenza A virus [17,18,19], the large (L) protein [20,21] and the core antigen (HBcAg) [22] from hepatitis B virus, and the gp41protein from the human immunodeficiency virus (HIV) [23,24,25]. The inhibitory properties of peptides have been reported for SARS-CoV, targeting the S protein [26,27], N protein [28], and main protease (M^pro^) [29]. Regarding SARS-CoV-2, recently, the use of peptide-based inhibitors has been reported for the S protein [30,31], M^pro^ [32], and PL^pro^ [33].

The Bothropstoxin-I (BthTX-I) is a myotoxin isolated from the venom of the *Bothrops jararacussu* snake that is homologous to the Phospholipase A_2_ (PLA_2_) [34]. Although not catalytically active, the C-terminal region of the toxin has shown antimicrobial effects. Peptides derived from the C-terminal region, including the p-BthTX-I and its disulfide-linked dimer, have been synthesized and showed considerable antimicrobial activity against gram-positive, gram-negative, and multi-drug resistance bacterial strains [34,35]. The serum degradation product obtained from this peptide, des-Lys^12^,Lys^13^-(p-BthTX-I)_2_, has shown greater antimicrobial activity than the disulfide-linked dimer [35]. In view of this biological potential, these peptides could be attractive molecules for the discovery and development of peptide-based therapies for infectious diseases, including COVID-19.

Herein, the synthesis and antiviral effects of the lysine linked dimer des-Cys^11^,Lys^12^,Lys^13^-(pBthTX-I)_2_K (**(pBthTX-I)_2_K**) and derivatives against SARS-CoV-2 are reported. To shed light on the mechanism of action underlying the peptides’ antiviral activity, the PL^pro^ inhibitory activity of the peptides was assessed. The findings indicated that **(pBthTX-I)_2_K** and analogs are attractive in vitro cellular and enzymatic inhibitory activities, thereby suggesting that they are promising prototypes for the discovery of new peptide-based compounds as lead compounds against COVID-19.

## 2. Results and Discussion

### 2.1. **(p-BthTX-I)_2_K** and Analogs Inhibit SARS-CoV-2 Infection In Vitro

The synthesis and characterization of the peptides derived from the C-terminal region of BthTX-I (named as p-BthTX-I, sequence: KKYRYHLKPFCKK) were previously reported [34,35]. The peptide analogs showed attractive inhibitory activity against Gram-negative (*Escherichia coli*), Gram-positive (*Staphylococcus aureus*) bacteria, and *Candida albicans* [35]. The results indicated that pBthTX-I and its disulfide-linked dimer, (pBthTX-I)_2_ were micromolar inhibitors of both bacterial strains (MIC*^E. coli^* values of 16 and 4 µM, respectively; MIC*^S. aureus^* values of 64 and 32 µM, respectively). Both peptides were further evaluated against a panel of pathogenic bacteria [35], including multidrug-resistant strains, and presented antibacterial activity against nine out of twenty bacterial strains tested. It is worth mentioning that the peptide was active against methicillin-resistant *S. aureus* (MRSA) strains isolated from infected Brazilian patients [35]. The dimeric peptide was active against the same bacterial strains in the panel, and showed inhibitory activity against five additional strains, including multidrug-resistant *K. pneumoniae* strains (ATCC700603 and ATCC BAA1705). Moreover, the des-Lys^12^,Lys^13^-(p-BthTX-I)_2_ peptide exhibited greater antimicrobial activity than the disulfide-linked dimer (p-BthTX-I)_2_ [35]. In another analog, the Cys residue was replaced with Lys (**(p-BthTX-I)_2_K**) and the peptide was crowned in α- and ε-amino groups [34,36]. The modified peptide showed similar or superior antibacterial activity to the original peptide (data in submission). These findings suggested that non-toxic dimeric peptides derived from the C-terminal region of BthTX-I are attractive bioactive compounds as candidates for infectious disease. Therefore, the inhibitory activities of these peptides were assessed against SARS-CoV-2.

A high-content screening (HCS) assay was developed to assess the effects of inhibitor candidates on infection and cytotoxicity on Vero cells infected with an isolate of SARS-CoV-2 [37]. Of note, substantial variability in the infection rates was observed during assay standardization, thereby impacting the readouts and assay sensitivity. The variability in the infection rates is related to the expression level of TMPRSS2 in Vero cells [38]. TMPRSS2 is the molecular receptor that the virus uses to prime the S protein, of which the expression level is considerably low in this cell line. Because of this limitation in the assay, chloroquine (CQ, EC_50_ = 7 µM; CC_50_ = 76 µM) and brequinar (BREQ, EC_50_ = 0.4 µM; CC_50_ > 10 µM) were used as positive controls as these drugs show selective antiviral activity in Vero cells in vitro (SI values of 11 and >25, respectively). The antiviral activity of these reference drugs was confirmed by RT-qPCR (Figure S1, Supplementary Material). Moreover, the peptide Hylin a1 (**Hy-a1**), a toxin from the frog *Hypsiboas albopunctatus* [39], was added to the study as an alternative positive control for inhibition and cytotoxicity (EC_50_ = 4 µM; CC_50_ = 81 µM).

**Figure 1:**
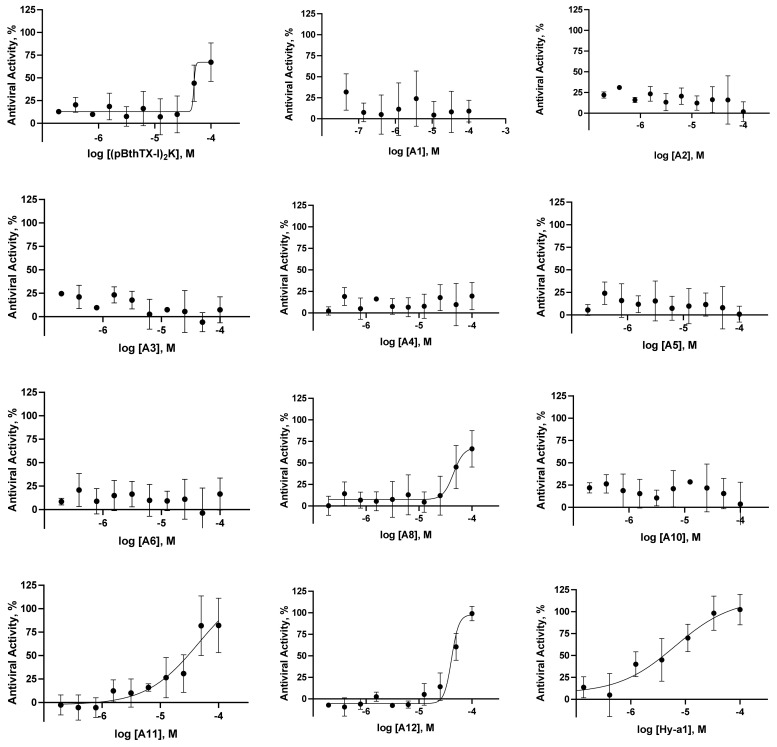

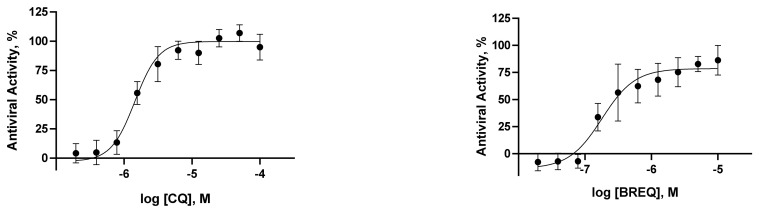
SARS-CoV-2 antiviral concentration–response curves for the peptides **(pBthTX-I)_2_K**, **A1**–**6**, **A8**, and **A10**–**12** (**Hy-a1**), chloroquine (**CQ**), and brequinar (**BREQ**) were used as positive controls for inhibition). The fitted EC_50_ values of the active inhibitors are the average of two independent experiments.

We assessed the inhibitory activities of the **(p-BthTX-I)2K** and analogs, including a tetrameric derivative, a D-isomer, and a series of analogs designed based on the alanine scanning strategy [40], to verify the contribution of the residues to the antiviral activity (Figure 1 and Table 1). The peptides showed inhibition of viral infection in the range of 4 to 70% at 100 µM (Table S1). Next, we assessed the EC_50_ values of the **(p-BthTX-I)2K** and analogs (Table 1). The natural peptide **(p-BthTX-I)_2_K** (EC_50_ = 65 µM), **A8** (EC_50_ = 67 µM), and the tetrameric analog (**A11**, EC_50_ = 51 µM) showed similar inhibitory potency. Representative images of SARS-CoV-2 infected Vero cells treated with the peptides are shown in Figure S2. We designed the tetrameric peptide, containing the Lys_2_Lys (K_2_K) as a dendrimeric core, to improve the biological activity [41]. Santos-Filho et al. [35] showed that the dimerization of the (BthTX-I) enhanced antimicrobial activity. However, in the SARS-CoV-2 assay, the tetrameric analog showed akin inhibitory potency to **(p-BthTX-I)_2_K**, suggesting that the dendrimeric core had little impact on the inhibitory property of the natural peptide. The d-isomer peptide (**A12**, EC_50_ = 28 µM) showed slightly enhanced antiviral activity within the series, with inhibitory potency in the low micromolar range. The peptide analogs **A1**–**6** and **A10** showed poor anti-SARS-CoV-2 activity in vitro (EC_50_s > 100 µM). These data indicated that the replacement with Ala residues on **(p-BthTX-I)_2_K** had no significant impact on the antiviral activity of the natural peptide. It is important to note that the cytotoxic activity of the **(p-BthTX-I)2K** and Ala analogs were low even at the highest concentration tested (CC_50_ > 100 µM), except for the tetrameric analog (**A11**), which showed a cytotoxic effect at the low micromolar range (CC_50_ = 2 µM). In sum, **(p-BthTX-I)2K, A8,** and **A12** derivatives showed promising antiviral and selective activity.

### 2.2. **(p-BthTX-I)_2_K** and Analogs Are Potent SARS-CoV-2 PL^pro^ Inhibitors

**Figure 2:**
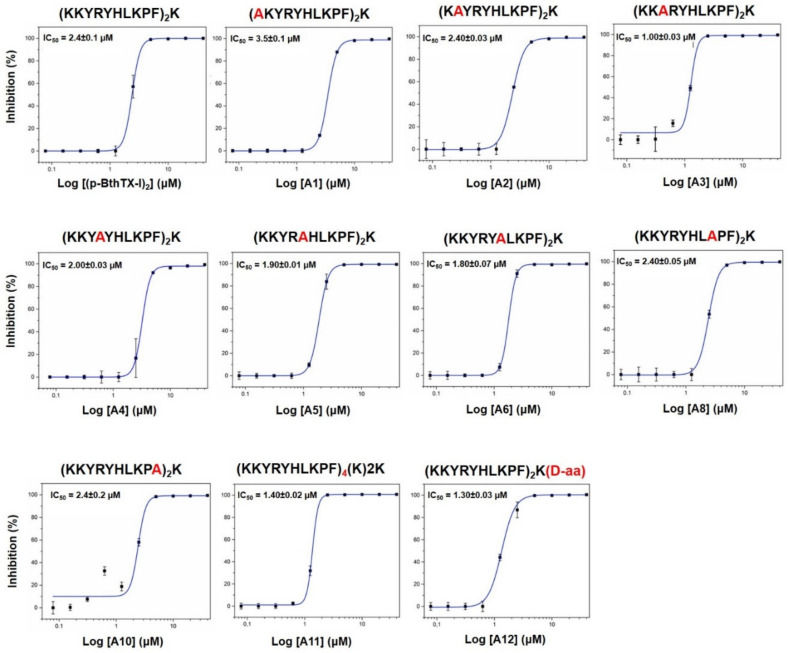
Representative concentration–response inhibition curves against PL^pro^ from SARS-CoV-2. The assessed IC_50_ values for the peptides **(pBthTX-I)_2_K**, **A1**–**6**, **A8**, and **A10**–**12** were determined from two independent experiments.

Aiming to identify a molecular target for **(pBthTX-I)_2_K** and analog antiviral activity and inspired by a previous report on peptides as inhibitors of viral proteases [15], we assessed the inhibitory effects of the synthetic peptides against both M^pro^ and PL^pro^ proteases from SARS-CoV-2 (Figure S3). The peptides showed no detectable effects on M^pro^ activity (IC_50_ > 10 μM). By contrast, **(pBthTX-I)_2_K** and analogs considerably decreased the PL^pro^ activity at 10 µM (>95% reduction) (Table S1). To better understand the structural requirements underlying the inhibitory activity, we assessed the PL^pro^ inhibitory potencies of the **(pBthTX-I)_2_K** analogs (Table 1 and Figure 2). The systematic amino acid substitutions were focused on the replacement of each positively charged (**A1**, **A2**, **A4**, **A6**, and **A8**) or aromatic (**A3**, **A5**, and **A10**) residues of the lead peptide. The **Hy-a1** toxin [39] was added to the study as a control to investigate unspecific interaction with the PL^pro^. **Hy-a1** showed poor inhibition of the PL^pro^ activity (13% of inhibition at 10 µM) (Figure S3 and Table S1), demonstrating that the enzyme was not subject to unspecific inhibition. The concentration–response curves showed a typical sigmoidal profile, indicating that the inhibition of PL^pro^ activity increased as a function of peptides concentration (Figure 2). Our findings indicated that the Ala-substituted analogs showed comparable inhibition profiles and IC_50_ values (IC_50_s = 1.25–3.5 μM) to the lead peptide **(pBthTX-I)_2_K**. Notably, the change between 0 and 100% of PL^pro^ activity is observed in less than a 10-fold change in inhibitor concentration, suggesting that the binding of the inhibitors to the subsites of the PL^pro^ catalytic cleft is not an independent process. Indeed, the binding of a substituent of the ligand at one subsite can positively or negatively impact the binding at other subsites. This phenomenon has been characterized as subsite cooperativity and has been reported for members of the serine, cysteine, and aspartic protease families [42]. The interdependence of binding at each subsite of the catalytic site in papain, a PL^pro^ homolog, has been shown previously [43]. In papain, the interdependence of subsites arose from the entropic cost of forming the enzyme–substrate transition state. As favorable contacts were added successively to a ligand, the entropic penalty associated with each decreased and the free energy expressed approached the incremental interaction energy. In line with this, the size of the peptides investigated herein (>2000 Da, Table 1) contributed to both probing the subsites more distant from the catalytic center and decreasing the entropic penalty with successive additions of favorable polar and hydrophobic interactions.

Moreover, similar to cooperativity in systems containing multiple ligand binding sites, cooperativity in monomeric single-site enzymes can be explained by protein conformational changes [44]. PL^pro^ flexibility and conformational changes were investigated in the ligand-free and ligand-bound states to characterize the overall protein dynamics [45,46]. These studies indicated that residues of the blocking loop 2 (BL2) play major roles in the unbinding pathways. Specifically, residues Asn267, Gln269, and most importantly Tyr268, account for most of this motion, which resembles the opening and closing of the loop [45]. Simulations showed that the BL2 loop in SARS-CoV-2 PL^pro^ is highly stabilized by ligand binding [46]. Of note, the sidechain and backbone rotation of Tyr268 is reduced by a hydrogen bond and strong van der Waals interactions with the ligand. In sum, cooperativity in monomeric enzymes has been reported in only a small number of cases, therefore a comprehensive investigation of this phenomenon underlying the PL^pro^ inhibition using new data would be helpful; however, such an analysis is beyond the scope of this work.

To provide an in-depth investigation of the binding properties of the peptides to PL^pro^, we selected representative inhibitors (**(pBthTX-I)_2_K**, **A1**–**6**, **A11**, and **A12**) for microscale thermophoresis (MST) analyses, an immobilization-free technology [47]. In this assay, the movement of molecules in a temperature gradient is monitored by a fluorescent probe covalently bound to the protein. The relative change of the fluorescence detected as a function of the temperature gradient applied is used to determine the binding affinities between the protein and the small molecules under investigation. The assessed *K*_d_ values for the representative peptides were in the low micromolar range (*K*_d_ = 0.9–7 µM) (Table 1 and Figure S4), which agreed with the determined potency values. These interaction data indicated that the peptides bind directly to the enzyme and confirmed that PL^pro^ is a potential target protein underlying the antiviral activity.

**Figure 3:**
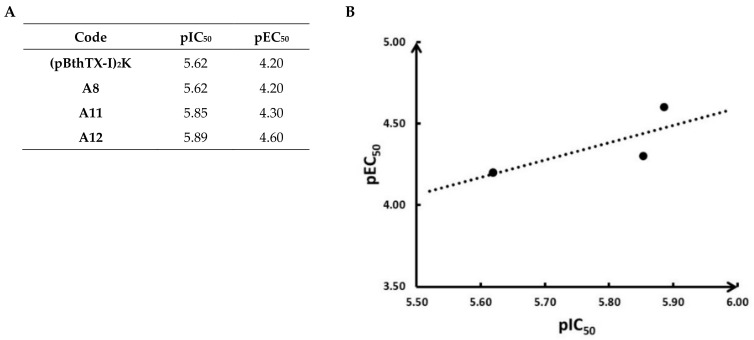
(**A**) Calculated −logEC_50_ (pEC_50_) and −logIC_50_ (pIC_50_) values of peptide analogs **(pBthTX-I)_2_K**, **A8**, **A11**, and **A12**. (**B**) Plot of the correlation between the pEC_50_ and pIC_50_ values (*r*^2^ = 0.6).

A preliminary structure–activity relationship (SAR) analysis suggested that the peptides’ binding potencies did not rely on a specific residue of the sequence but the contribution of the whole structure to the inhibitory activity. This is verified by the activity of the d-isomer analog (**A12**). Due to its D-amino acid, the peptide has different structural conversion, but the mirror symmetry could allow it to specifically bind with reasonable affinity to PL^pro^. Additionally, d-chiral analogs are more active peptides because proteases are unable to hydrolyze the d-amino acids [48]. The IC_50_ values of the peptide analogs are close to the ones recently reported for other SARS-CoV-2 PL^pro^ inhibitors as lead compounds, such as the naphthalene-based derivatives GRL0617 (IC_50_ of 2.2 μM) [49] and compound 6 (IC_50_ of 5 μM) [50]. It is worth mentioning that we observed a reasonable correlation between the SARS-CoV-2 antiviral effect (EC_50_ values) and the inhibitory activity against PL^pro^ (IC_50_ values) (Figure 3). This finding corroborates that one of the mechanisms of action underlying the SARS-CoV-2 inhibitory activity might be via PL^pro^ inhibition.

### 2.3. **(pBthTX-I)_2_K** Analog Blocks the Entry of the Substrate toward the Protease Catalytic Cleft

**Figure 4:**
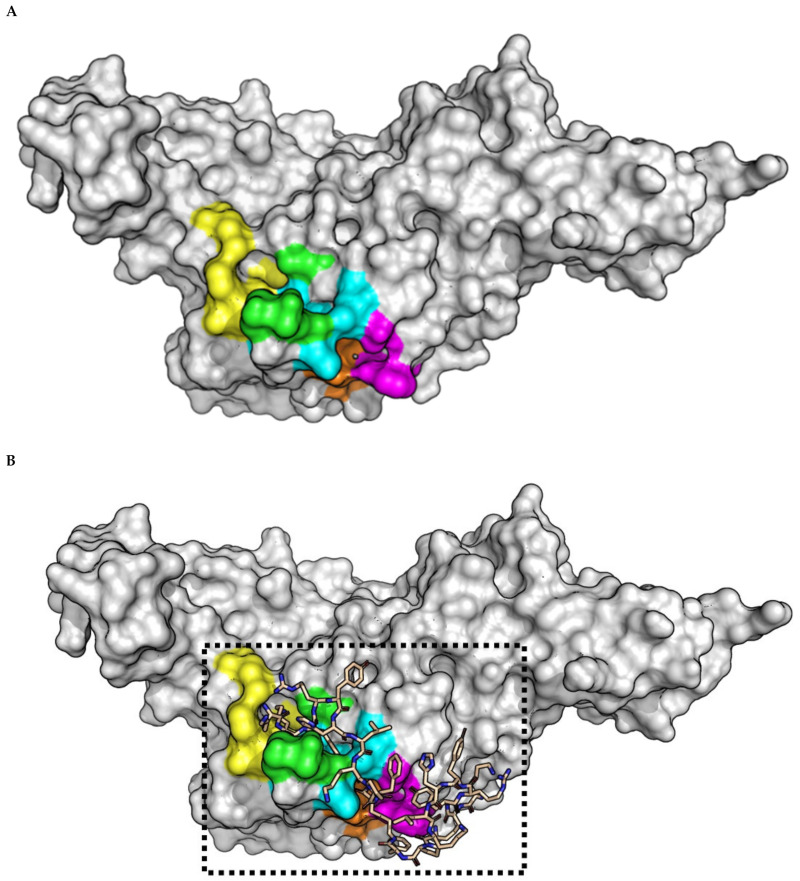

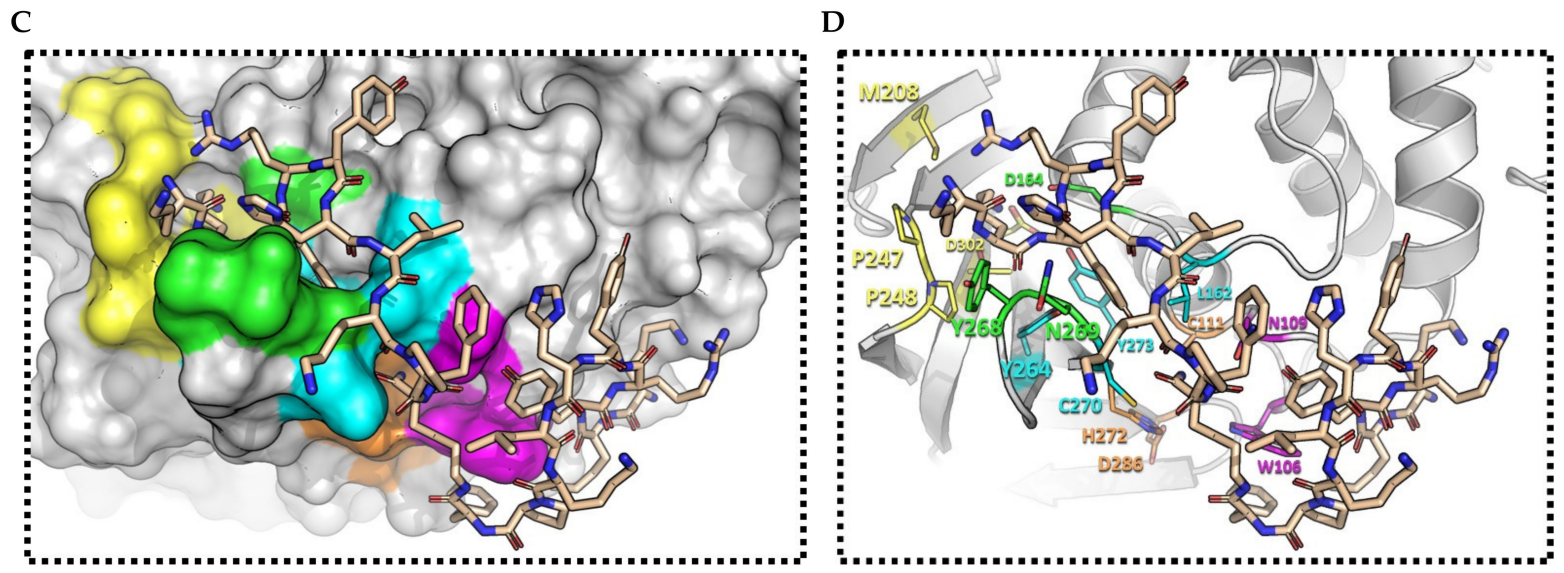
(**A**) SARS-CoV-2 PL^pro^ crystallographic model (PDB ID: 6WX4). The catalytic (orange) and subsites S2 (cyan), S3 (green), S4 (yellow), and oxyanion hole (magenta) residues are indicated. (**B**) Top-ranked binding mode of **(pBthTX-I)_2_K** (wheat sticks) into SARS-CoV-2 PL^pro^ binding site. (**C**) Close-up view of the Conolly surface model of SARS-CoV-2 PL^pro^ structure in complex with **(pBthTX-I)_2_K** (binding energy = −5.2 kcal/mol). (**D**) Detailed view of **(pBthTX-I)_2_K** bound to SARS-CoV-2 PL^pro^ (cartoon model) catalytic site (subsites residues are colored as in (**A**). Residues Y268, N269 (S3 subsite), and C270 (S2 subsite) lay on the BL2 loop (265-TGNYQCG-271).

To shed light on the structural determinant underlying the inhibitory activity of the peptides, we selected the natural peptide **(pBthTX-I)_2_K** as a representative compound of the series and modeled its binding mode to PL^pro^ from SARS-CoV-2 (Figure 4). The enzyme recognizes and cleaves the LXGG motif to release the nsp1, nsp2, and nsp3 viral proteins [32]. In contrast to M^pro^, PL^pro^ recognizes a greater diversity of substrates [51]. For instance, S1 and S2 subsites bind glycine residues only, whereas aromatic, hydrophobic, and positively charged groups are tolerated in the S3 pocket. The S4 subsite recognizes hydrophobic residues and has a preference for leucine [32] (Figure 4A,B).

The modeled binding mode of **(pBthTX-I)_2_K** indicated that the inhibitor is in close contact with key amino acid residues involved in the catalysis (Figure 4C,D). Specifically, the inhibitor binds to the conserved amino acid residues in the S2 (Leu162, Cys270), S3 (Tyr268, Gln269), S4 (Met208, Asp302) pockets, and the oxyanion hole (Trp106, Asn109) of PL^pro^, making extensive polar and hydrophobic interactions.

The peptide binding mode is stabilized by key hydrogen bonds to the side chain of Gln269 and the main chain of Gly271, as well as van der Waals interactions with the side chain of Tyr268. These residues belong to the BL2 loop (265-TGNYQCG-271) in the palm domain of PL^pro^, which is a crucial structural element for substrate recognition and inhibitor binding [12,51]. This binding mode indicated that **(pBthTX-I)_2_K** and its analogs possibly block the entry of the substrate toward the protease catalytic cleft, thereby inhibiting the catalytic activity of the enzyme. Moreover, these findings suggest that observed cooperativity within PL^pro^ subsites can be driven by either enthalpic/entropic contribution upon ligand binding or stabilization of protein conformations that show enhanced affinities for the ligands [44].

As **(pBthTX-I)_2_K** and **A3**, **A5**, **A6**, **A8**, **A11**, and **A12** analogs showed akin inhibitory potencies against PL^pro^ (Table 1), we hypothesized that this could be due to the presence of the many flexible bonds in the peptide structure [52]. Indeed, the inhibitor exhibited a considerable degree of freedom in the modeling studies, which contributed to the sampling of favorable conformational states for binding. The analysis of the top five binding modes of **(pBthTX-I)_2_K** indicated that the poses had binding energy values that varied from −4.7 to −5.2 kcal/mol with root-mean-square deviation (rmsd) values of <3.7 Å between each pose and the top-ranked binding modes (Figure 4A,B and Figure S5). These results suggest that the top five predicted poses for **(pBthTX-I)_2_K** showed slightly different binding modes to PL^pro^ but similar binding energies (Figure S5). Accordingly, the structural flexibility of the peptides enabled alternative binding poses that have compensated for the binding energy losses caused by the Ala replacement.

## 3. Conclusions

In conclusion, we showed that the non-toxic dimeric peptide from the bothropstoxin-I **(pBthTX-I)_2_K** and analogs are active against SARS-CoV-2. The active peptides showed antiviral activity in the micromolar range, low cytotoxic effects, and attractive selectivity indexes. Importantly, the peptide analogs showed inhibitory potencies against SARS-CoV-2 PL^pro^ in the same range of activity as observed in the phenotypic assay, suggesting that the protease could be one of the molecular targets underlying the antiviral activity. Our findings represent a valuable resource in the exploration of new molecules as prototypes for drug discovery and development against SARS-CoV-2 infections.

## 4. Materials and Methods

### 4.1. Peptides Synthesis

The peptides were manually synthesized by solid-phase using the Fmoc (9-fluorenylmethyloxycarbonyl) protocol [53]. Briefly, deprotection of the Fmoc group was performed in 20% 4-methylpiperidine in dimethylformamide (DMF). Fmoc-amino acids were coupled with 2 times excess using diisopropylcarbodiimide (DIC)/*N*-hydroxybenzotriazole (HOBt) in DMF (*N*,*N*-dimethylformamide). The C-terminal dimers were obtained as previously described by Lorenzón et al. [54], whereby Fmoc-Lys(Fmoc)-OH was attached to the Rink resin and, after α- and ε-Fmoc group deprotection, the two chains of peptides were simultaneously elongated. The tetrameric peptide was obtained by the addition of two others Fmoc-Lys(Fmoc)-OH, deprotection, and simultaneously elongation. The cleavage of the peptide from the resin was performed for 2 h using 95% TFA, 2.5 TIS (triisopropylsilane), and 2.5 water, at a ratio of 10 mL/g resin. Following this procedure, the crude peptides were precipitated with chilled ethyl ether, separated from soluble non-peptide material by centrifugation, and lyophilized. Purification of the synthetic peptides was performed in a semi-preparative mode using a C18 reverse-phase column (AAPPTEC 1.0 × 25 cm). The purity was determined on a C_18_ reverse-phase (0.46 × 25 cm) analytical column (Agilent, Santa Clara, CA, USA). The solvents were 0.045% TFA in deionized water and 0.036% TFA in acetonitrile with 1 mL/min with a linear gradient of 5–95% solvent B (0.036% (*v*/*v*) TFA/acetonitrile) for 30 min. The identity of the peptide was confirmed by mass spectrometry in positive-ion mode ESI. The Fluorescence Resonance Energy Transfer (FRET) peptide Abz-T-L-K-G-G-A-P-I-K-Q-EDDnp (Abz = *ortho*-aminobenzoic acid and EDDnp = *N*-[2,4-dinitrophenyl]-ethylenediamine) used as a PL^pro^ substrate corresponds to cleavage at the nsp 2/3 site of the virus polyprotein. This peptide (PL^pro^ substrate) was synthesized by the Fmoc-procedure [55] using solid-phase synthesis and the automated bench-top simultaneous multiple solid-phase peptide synthesizer (PSSM 8 system from Shimadzu, Kyoto, Japan). The final peptides were deprotected in TFA and purified by semipreparative HPLC using an Econosil C-18 column (10 µm, 22.5 × 250 mm) and a two-solvent system: (A) Trifluoroacetic acid (TFA)/H_2_O (1:1000) and (B) TFA/acetonitrile (ACN)/H_2_O (1:900:100). The column was eluted at a flow rate of 5 mL/min with a 10 (or 30) –50 (or 60)% gradient of solvent B over 30 or 45 min. Analytical HPLC was performed using a binary HPLC system from Shimadzu with an SPD-10AV Shimadzu UV-vis detector, coupled to an Ultrasphere C-18 column (5 µm, 4.6 × 150 mm), which was eluted with solvent systems A1 (H_3_PO_4_/H_2_O, 1:1000) and B1 (ACN/H_2_O/H_3_PO_4_, 900:100:1) at a flow rate of 1 mL/min and a 10–80% gradient of B1 over 20 min. The HPLC column eluates were monitored by their absorbance at 220 nm. The molecular weight and purity of the peptides were checked by MALDI-TOF mass spectrometry (Bruker Daltons) or electron spray LC/MS-2020 (Shimadzu, Kyoto, Japan). Stock solutions of peptides were prepared in DMSO, and the concentration was measured spectrophotometrically using a molar extinction coefficient of 17.300 M^−1^cm^−1^ at 365 nm from EDDnp. LC/MS-2020 identified the cleaved bonds. The peptides’ integrity was assessed by analytical HPLC using a binary HPLC system from Shimadzu (Kyoto, Japan) with an SPD-10AV Shimadzu UV-Vis detector, coupled to an Ultrasphere C-18 column (5 µm, 4.6 × 250 mm), which was eluted with solvent systems A1 (TFA/H2O, 1:1000) and B1 (ACN/H2O/TFA, 900:100:1) at a flow rate of 1.0 mL/min and a 5–80% gradient of B1 over 10 min. The HPLC column eluates were monitored by their absorbance at 220 nm. The molecular weight was checked by mass spectrometry 2020 (Shimadzu, Kyoto, Japan) (Figure S6).

### 4.2. Phenotypic Screening Assay

All procedures involving the SARS-CoV-2 virus were performed in the biosafety level 3 laboratory at the Institute of Biomedical Sciences of the University of São Paulo. SARS-CoV-2 (HIAE-02: SARS-CoV-2/SP02/human/2020/BRA, GenBank Accession No. MT126808.1) was isolated from a nasopharyngeal sample of a confirmed COVID-19 patient in São Paulo, Brazil [56] The virus was passaged twice in the Vero cell line (Vero CCL-81, obtained from the Culture Collection Laboratory, Instituto Adolfo Lutz, São Paulo, Brazil) maintained in DMEM High Glucose (Sigma-Aldrich, St. Louis, MO, USA) supplemented with 2% heat-inactivated Fetal Bovine Serum (FBS; Thermo Fisher Scientific, Waltham, MA, USA) and 100 U/mL of penicillin and 100 μg/mL of streptomycin (Thermo Fisher Scientific, Waltham, MA, USA). Cells were maintained at 37 °C with 5% CO_2_. The supernatant collected from infected cell tissue culture was stored in aliquots at −80 °C and the viral titer was determined by plaque assay in Vero CCL-81. Briefly, 1 × 10^5^ Vero CCL-81 cells were seeded on each well of a 24-well plate in DMEM High Glucose supplemented with 10% FBS at 37 °C with 5% CO_2_. After 24 h, the entire medium was removed and replaced by 400 µL of medium without supplementation and containing serial dilutions of SARS-CoV-2. The plates were incubated for 1 h at 37 °C with 5% CO_2_ for virus adsorption. Then, the medium was removed and replaced with 500 µL of DMEM High Glucose (2% FBS) containing 2% of carboxymethyl cellulose, and the plates were incubated for another 72 h. After that, the medium was removed, plates were fixed with 4% paraformaldehyde in PBS (*m*/*v*) pH 7.4 for 15 min, and stained with crystal violet 1% in 10% ethanol (*m*/*v*/*v*) for 5 min. The number of plaques was visually assessed, counted, and the virus titer was calculated as plaque-forming units (PFU)/mL.

For inhibitor screening phenotypic assays, 6000 Vero cells were seeded on each well of a 96-well assay plate (Greiner Bio-One, Frickenhausen, Germany in 120 µL of DMEM High Glucose (Sigma-Aldrich, St. Louis, MO, USA) supplemented with 10% heat-inactivated FBS (Thermo Fisher Scientific, Waltham, MA, USA), 100 U/mL of penicillin, and 100 μg/mL of streptomycin (Thermo Fisher Scientific, Waltham, MA, USA) at 37 °C, 5% CO_2_ for 24 h. Next, the medium was removed and 60 µL of DMEM High Glucose (Sigma-Aldrich, St. Louis, MO, USA) was added to each well. Serially diluted compounds were manually transferred into a polypropylene 96-well plate (Greiner Bio-One, Frickenhausen, Germany) containing sterile phosphate-buffered saline (PBS) pH 7.4, for a final dilution factor of 33.3. Then, 30 µL of each well in the compounds plate was transferred to the cell-containing assay plate, followed by the addition of SARS-CoV-2 viral particles to the cells at the multiplicity of infection (MOI) 0.1 in 30 µL of DMEM High Glucose per well. DMSO-treated infected cells and DMSO-treated non-infected cells were used as controls. The assay plate was incubated for 1 h at 37 °C, 5% CO_2_ for virus adsorption, followed by the addition of 60 µL of DMEM High Glucose supplemented with 6% FBS per well. The final concentrations in the assay plate were 0.5% DMSO and 2% FBS (*v*/*v*). After 33 h of incubation, the plates were fixed in 4% paraformaldehyde in PBS pH 7.4 and subjected to indirect immunofluorescence detection of viral cellular infection. After washing twice with PBS pH 7.4, plates were blocked with 5% bovine serum albumin (BSA) (Sigma-Aldrich, St. Louis, MO, USA) in PBS (BSA-PBS) for 30 min at room temperature and washed twice with PBS. The infection was detected using an immunofluorescence assay. Hyperimmune serum from a convalescent COVID-19 Brazilian patient diluted 1:1000 in 5% BSA in PBS (*v*/*v*) was used as the primary antibody. After 30 min of incubation, wells were washed and a solution containing Alexa488 conjugated goat anti-human IgG (Thermo Fisher Scientific, Waltham, MA, USA) and 5 µg/mL of DAPI (4′,6 diamidino-2-phenylindole; Sigma-Aldrich, St. Louis, MO, USA) diluted 1:1000 in 5% BSA (*v*/*v*) was added to each well. After the final incubation for 30 min, the plates were washed twice with PBS and submitted to imaging in the Operetta High Content Imaging System (Perkin Elmer, Waltham, MA, USA) using a 20× magnification objective. Acquired images were analyzed in the software Harmony (Perkin Elmer, Waltham, MA, USA), version 3.5.2. Image analysis included the identification and counting of the Vero cells based on nuclear segmentation and viral infection based on the cytoplasmic staining as detected by the immunofluorescence assay. Automated cell segmentation and identification of SARS-CoV-2-infected cells were visually confirmed. The infection ratio (IR) was calculated as the ratio between the number of infected cells and the number of total cells counted in each well. The cell survival rate was calculated as the number of cells counted in each well divided by the average number of cells in the positive control (DMSO-treated infected cells) wells, multiplied by 100. The antiviral activity was determined by the normalization of the IR to the negative control (DMSO-treated infected and non-infected cells), as described. Concentration–response curves were plotted using the normalized activity and cell survival of each concentration with nonlinear regression analysis and the sigmoidal dose–response (variable slope) function using GraphPad Prism version 7.0 (GraphPad Software, San Diego, CA, USA). EC_50_ and CC_50_ values were determined by interpolation of unknowns to the fitted sigmoidal response curves and were defined as compound concentrations that reduce the infection ratio and cell survival by 50%, respectively, compared to non-treated infected controls of each compound. The data reported were obtained in two independent experiments. As a quality control, the Z’-factor [57] was determined for each plate, and only plates with Z’-factor > 0.50 were approved for analysis. The antiviral activity of reference compounds (CQ and Breq) was also confirmed by RT-qPCR. Briefly, 100 µL of the culture supernatant of infected Vero cells treated with the vehicle (0.5% DMSO, *v*/*v*), chloroquine, or brequinar at different concentrations, and non-infected Vero cells were collected and immediately submitted to RNA extraction using the MagMAX™ CORE Nucleic Acid Purification Kit (Thermo Fisher Scientific, Waltham, MA, USA) and the MagMAX™ Express-96 Deep Well Magnetic Particle Processor (Thermo Fisher Scientific, Waltham, MA, USA), following the manufacturer’s instructions. SARS-CoV-2 RNA quantification was determined by RT-qPCR using the AgPath-ID™ One-Step RT-PCR kit (Thermo Scientific, Waltham, MA, USA), following the manufacturer’s instructions, and primers and probe for the E gene: E_Sarbeco_F: ACAGGTACGTTAATAGTTAATAGCGT-3′, E_Sarbeco_P: FAM-ACACTAGCCATCCTTACTGCGCTTCG-QSY-3′ and E_Sarbeco_R: 5′-ATATTGCAGCAGTACGCACACA [58] in an Applied Biosystems 7500 Real-Time PCR (Thermo Fisher Scientific, Waltham, MA, USA) instrument. The determination of viral genome copy number was performed as described [56].

### 4.3. SARS-CoV-2 PL^pro^ Cloning, Expression, and Purification

The viral cDNA template (GenBank MT126808.1), kindly provided by Dr. Edison Durigon (University of São Paulo, São Paulo, Brazil), was synthesized using the SCRIPT One-Step RT-PCR kit (Cellco Biotec, São Carlos, Brazil) and random hexamers primers. Amplification of the nucleotide sequence coding for the PL^pro^ domain (residues 1564–1879 of SARS-CoV-2 orf1ab polyprotein) (GenBank: QIG55993.1) was performed by Polymerase Chain Reaction (PCR) using forward (5′-ATT**CCATGG**GCGAAGTGAGGACTATTAAGGTGTTTAC-3′) and reverse (5′-ATTG**CTCGAG**TGGTTTTATGGTTGTTGTGTAACT-3′) primers, with restriction sites for *NcoI* and *XhoI* indicated in bold. The reaction was carried out with FastPol HF DNA Polymerase (Cellco Biotec, São Carlos, Brazil). The PCR product was digested with *NcoI* and *XhoI* and cloned into pET28a (Novagen, Madison, WI, USA) in a frame with a C-terminal His-tag coding sequence.

The plasmids were used to transform *Rosetta 2 (DE3) E. coli* cells (Novagen, Madison, WI, USA), which were grown in Lysogen Broth (LB) medium, supplemented with 50 μg/mL kanamycin, and 34 μg/mL chloramphenicol at 37 °C until the OD_600_ reached 0.6. The protein expression was induced by the addition of 0.5 mM Isopropyl β-d-1-thiogalactopyranoside (IPTG) and 1 mM zinc sulfate (ZnSO_4_), for 16 h at 18 °C. Next, cells were harvested by centrifugation, and cell pellets were resuspended in lysis buffer (50 mM Tris-HCl pH 8.5, 150 mM NaCl, 10 mM imidazole, and 1 mM DTT). Cells were then lysed by sonication and centrifuged 15,000× *g* to clarify the supernatant.

The SARS-CoV-2 PL^pro^ was purified using an AKTA Purifier System (GE Healthcare, Boston, MA, USA). The first purification step was affinity chromatography using a HisTrap HP 5.0 mL column (GE Healthcare, Boston, MA, USA). The protein was eluted with an elution buffer (50 mM Tris-HCl pH 8.5, 150 mM NaCl, 250 mM imidazole, and 1 mM DTT) and after that, the second purification step was done through size-exclusion chromatography on a HiLoad Superdex 75 16/60 column (GE Healthcare, Boston, MA, USA) pre-equilibrated with 20 mM Tris-HCl pH 7.4, 100 mM NaCl, and 1 mM TCEP. The final protein sample was analyzed in SDS-PAGE 12.5% to confirm its purity (Figure S7). The protein was concentrated to 1.0 mg/mL followed by the addition of 5% glycerol, and samples were then flash-frozen and stored at −80 °C for activity assays. Concentration was determined spectrophotometrically in a Nanodrop 1000 spectrophotometer based on the theoretical extinction coefficient of 45,270 M^−1^.cm^−1^.

The cloning, expression, and purification of SARS-CoV-2 M^pro^ was conducted as described elsewhere [59].

### 4.4. Enzyme Inhibition Assays

The SARS-CoV-2 PL^pro^ inhibition assay was performed using the FRET-peptide Abz-TLKGG↓APIKEDDPS-EDDnp (↓ cleavage site) as reported in [60]. The assay was standardized with an enzyme concentration at 70 nM. The fluorescent substrate was used at 27 µM in an assay buffer containing 50 mM HEPES pH 7.5, 0.01% Triton X-100 and 5 mM DTT. The negative control was made with only DMSO (used to dilute the peptides) and the peptide **Hylin a1** [39] was used as a control of enzyme specificity. The protein was diluted in an assay buffer and incubated with 10 µM of each peptide, at 37 °C for 30 min. Then, the diluted substrate was added to the solution and the enzymatic activity was measured in the spectrofluorometer system Spectramax Gemini EM (Molecular devices, San Jose, CA, USA), with λ_ex_ = 320 nm and λ_em_ = 420 nm, at 37 °C every 30 s for 15 min. Time-dependent traces of the substrate fluorescence at different inhibitor concentrations are shown in Figure S8. At lower concentrations, some peptides showed a similar signal with equal intensity to the noise, indicating that the signal of the peptides did not exceed the basic noise at these concentrations. As a rule, the intensity of the signal displayed by the reaction must exceed the noise at least by a factor of two. Therefore, as the reaction was not detectable by the method at lower concentrations, the collected data with a signal-to-noise ratio < 2 or greater than the DMSO control were corrected to zero and treated as artifacts in the assay because no inhibition was observed at these concentrations.

The SARS-CoV-2 M^pro^ inhibition assay was carried out using the FRET-based substrate DABCYL-KTSAVLQ↓SGFRKME(EDANS)-NH2 in an assay buffer (20 mM Tris pH 7.3, 1 mM EDTA, 1 mM DTT) as reported in [59]. M^pro^ was used at a final concentration of 0.14 μM. Before reactions, the enzyme was incubated in an assay buffer at 37 °C for 10 min. Substrate concentration was maintained at 20 µM, and peptides were tested at a single concentration of 10 µM. The initial velocity was derived from the slope of the linear phase of each time-course reaction. The results were analyzed using OriginPro 9.0 Software (Origin Lab, Northampton, MA, USA). Fluorescence measures were performed in SpectraMax Gemini EM Microplate Reader with λ_exc_/λ_emi_ of 360/460 nm, every 30 s over 60 min at 37 °C. All assays were performed in triplicates.

The peptides that inhibited the SARS-CoV-2 PL^pro^ activity in more than 80% at 10 µM were assayed in a concentration-dependent manner to determine their half-inhibitory concentrations (IC_50_). Enzyme and substrate concentrations were maintained at 70 nM and 27 µM concentrations, respectively, and peptides were 2-fold serially diluted (from 40 µM to 0.079 µM). To verify the peptides’ inhibition profile as well as the replicability and reproducibility of the assay, we determined the IC_50_ values from two independent experiments (Figure S9). The results were analyzed using OriginPro 9.0 Software (Origin Lab, Northampton, MA, USA) and the IC_50_ values for each peptide were determined using the Hill function fitting.

### 4.5. Binding Assays (MicroScale Thermophoresis—MST)

Microscale thermophoresis (MST) was used to measure the affinity between the purified PL^pro^ and the peptide inhibitors. PL^pro^ was labeled on the Monolith His-Tag Labeling Kit RED-tris-NTA 2nd Generation as per the manufacturer’s instructions. Labeled PL^pro^ was kept at a constant concentration (100 nM), while the concentration of the nonlabelled peptides varied from 0.015 to 500 μM. The assay was carried out in 50 mM HEPES pH 7.5, 100 mM NaCl, 2 mM DTT, and DMSO 5%. The samples were loaded into Monolith NT.115 Standard Treated Capillaries and the MST analysis was carried out using the laser beam at 40% potency, in the Monolith^®^ NT.115 instrument (Nanotemper Technologies, München, Germany). All measurements were carried out in triplicate. The dissociation constant (*K*_d_) was obtained by fitting the binding curve with the Hill function, using OriginPro 9.0 Software (Origin Lab, Northampton, MA, USA).

### 4.6. Molecular Modeling

AutoDock Vina software [61] was used to perform molecular docking. SARS-CoV-2 PL^pro^ three-dimensional structure was retrieved from RCSB PDB [62] (PDB ID: 6WX4) [33]. The ligand bound to the catalytic site was used as a reference for the binding site definition. The 3D structure of the **(pBthTX-I)_2_K** peptide was prepared for docking by a minimization step, using Chem3D^®^ from PerkinElmer Informatics (Waltham, MA, USA) (gradient norm less than 0.010). The partial charges assignment was computed with the Gasteiger charge method, using AutoDockTools-1.5.6 [63]. The grid calculation was set using default parameters and centered at X = 8.143, Y = −26.919, and Z = −31.882. The search box was defined according to the 6WX4 ligand binding site using AutoDockTools-1.5.6 and set as 26 Å × 26 Å × 36 Å. Exhaustiveness was set to 10, with a maximum number of binding modes set to 15. The binding affinities of the top **(pBthTX-I)_2_K** poses bound to SARS-CoV-2 PL^pro^ were used to identify the binding mode that best correlated with the assessed inhibitory potency.

## Figures and Tables

**Table 1:** Inhibitory activities of **(pBthTX-I)_2_K** and analogs against SARS-CoV-2 and PL^pro^. The substituted Ala residue on the sequence of each peptide is indicated as red.

Code	Peptide Sequence	MW (Da)	EC_50_ (µM)	CC_50_ (µM)	SI	IC_50_ (µM)	*K*_d_ (µM)
**(pBthTX-I)2K**	(KKYRYHLKPF)_2_K	2868	65 ± 35	>100	>1.5	2.4 ± 0.1	0.9 ± 0.1
**A1**	(**A**KYRYHLKPF)_2_K	2754	>100	>100	n.d.	3.5 ± 0.1	6 ± 3
**A2**	(K**A**YRYHLKPF)_2_K	2754	>100	>100	n.d.	2.40 ± 0.03	7 ± 3
**A3**	(KK**A**RYHLKPF)_2_K	2684	>100	>100	n.d.	1.00 ± 0.03	6 ± 4
**A4**	(KKY**A**YHLKPF)_2_K	2698	>100	>100	n.d.	2.00 ± 0.03	3 ± 1
**A5**	(KKYR**A**HLKPF)_2_K	2684	>100	>100	n.d.	1.90 ± 0.01	3 ± 2
**A6**	(KKYRY**A**LKPF)_2_K	2736	>100	>100	n.d.	1.80 ± 0.07	5 ± 3
**A8**	(KKYRYHL**A**PF)_2_K	2754	67 ± 32	>100	>1.5	2.40 ± 0.05	n.d.
**A10**	(KKYRYHLKP**A**)_2_K	2716	>100	>100	n.d.	2.4 ± 0.2	n.d.
**A11**	(KKYRYHLKPF)_4_(K)_2_K	5848	51 ± 40	2.0 ± 0.4	0.04	1.40 ± 0.02	1.0 ± 0.2
**A12**	(KKYRYHLKPF)_2_K(d-aa)	2868	28 ± 14	58 ± 5	2	1.30 ± 0.03	1.6 ± 0.3
**CQ**	--		7 ± 5	76 ± 26	11	n.d.	n.d.
**BREQ**	--		0.4 ± 0.3	>10	>25	n.d.	n.d.
**Hy-a1**	IFGAILPLALGALKNLIK-NH_2_	1865	4 ± 3	81 ± 58	20	>10	n.d.

MW = molecular weight; EC_50_ = effective concentration; CC_50_ = cytotoxic concentration; SI = selectivity index (CC_50_/EC_50_); CQ = chloroquine; BREQ = brequinar n.d. = not determined.

## Data Availability

The data that support the findings of this study are available from the corresponding author upon reasonable request.

## Supplementary Materials

The following are available online, Table S1: Inhibitory activity of (pBthTX-I)2K and analogs against SARS-CoV-2 and PLpro at 100 and 10 µM, respectively, Figure S1: RT-qPCR quantification of antiviral activity of reference drugs against SARS-CoV-2, Figure S2: Representative images of in vitro antiviral activity of peptides against SARS-CoV-2-infected Vero cells, Figure S3: Single concentration inhibition screening of peptide analogs, Figure S4; Kd values determination by microscale thermophoresis, Figure S5: Predicted binding modes of (pBthTX-I)2K to PLpro (top 2–5 poses), Figure S6: Peptides’ integrity assessment, Figure S7: SARS-CoV-2 PLpro purification, Figure S8: Representative time-dependent traces of the substrate fluorescence at different inhibitor concentrations, Figure S9: IC50 plots of representative peptides collected in November 2020 and June 2021.

## Sample Availability

Samples of the compounds **(pBthTX-I)_2_K**, **A1**–**6**, **A8**, and **A12** are available from the authors.
