# Supplementary material for: Non-Toxic Dimeric Peptides Derived from the Bothropstoxin-I Are Potent SARS-CoV-2 and Papain-like Protease Inhibitors
Source: Molecules. 2021 Aug 12;26(16):4896. doi: 10.3390/molecules26164896 (PMC8401042; doi:10.3390/molecules26164896)
Supplement: Supplementary file 1 [file molecules-26-04896-s001.zip › molecules-1179729-supplementary.pdf]

Non-toxic dimeric peptides derived from the Bothropstoxin-I are potent SARS-CoV-2 and Papain-Like Protease inhibitors

Marjorie C. L. C. Freire,[1] Gabriela D. Noske,[1] Natália V. Bitencourt,[2] Paulo R. S. Sanches,[2] Norival A. Santos-Filho,[2] Victor O. Gawriljuk,[1] Eduardo P. de Souza,[3] Victor H. R. Nogueira,[1] Mariana O. de Godoy,[1] Aline M. Nakamura,[1] Rafaela S. Fernandes,[1] Andre S. Godoy,[1] Maria A. Juliano[4], Bianca M. Peres,[5] Cecília Gomes Barbosa,[5] Carolina B. Moraes,[6] Lucio H. Freitas-Junior,[5] Eduardo M. Cilli,[2] Rafael V. C. Guido,[1] Glaucius Oliva[1*]*

[1] São Carlos Institute of Physics, University of Sao Paulo, Avenida João Dagnone, 1100, São Carlos, SP 13563-120, São Carlos, Brazil.

[2] Department of Biochemistry and Organic Chemistry, Institute of Chemistry, São Paulo State University, SP, Brazil.

[3] Department of Genetics and Evolution, Federal University of São Carlos, São Carlos, 13565-905 – São Paulo – Brazil

[4] The Sao Paulo School of Medicine, Federal University of São Paulo

[5] Department of Microbiology, Institute of Biomedical Sciences, University of Sao Paulo, Av. Prof. Lineu Prestes, 1374, Sao Paulo-SP 05508-900, Brazil.

[6] Department of Pharmaceutical Sciences, Federal University of São Paulo, Rua São Nicolau, 210, Diadema – SP, 09913-030, Brazil.

(*) Joint corresponding authors

Rafael V. C. Guido
Physics Institute of São Carlos, University of São Paulo
Av. Joao Dagnone, 1.100, Jd. Santa Angelina, 13563-120, Sao Carlos-SP, Brazil
Phone: +55 16 3373-8673
e-mail: rvcguido@usp.br
ORCID: http://orcid.org/0000-0002-7187-0818

Glaucius Oliva
Physics Institute of São Carlos, University of São Paulo
Av. Joao Dagnone, 1.100, Jd. Santa Angelina, 13563-120, Sao Carlos-SP, Brazil
Phone: +55 16 3373-6664
e-mail: oliva@ifsc.usp.br
ORCID: https://orcid.org/0000-0003-2719-0302

TABLE OF CONTENTS

Figure S1. RT-qPCR quantification of antiviral activity of reference drugs against SARS-CoV-2. Viral genome-equivalents in culture supernatants after 48 h of infection in the presence of vehicle (0.5% DMSO, light purple), 50 µM and 0.78 µM chloroquine (salmon) and 5 µM and 80 nM brequinar (green). The mean and standard error of the mean values from three independent experiments are shown…... S2

Figure S2. *In vitro* antiviral activity of peptides against SARS-CoV-2-infected Vero cells in the phenotypic screening assay. Representative images of infected cells treated with vehicle (0.5% DMSO, top left), 12.5 µM chloroquine (CQ, middle left), 100 µM (pBthTX-I)$_2$K (middle right), 100 µM A8 (bottom left) and 25 µM A12 (bottom right). SARS-CoV-2 infection is shown in green, as detected by indirect immunofluorescence assay (IFA) using an anti-S protein serum. Vero cell nuclei are shown in blue. Non-infected cells are shown as IFA control……………..………….. S3

Figure S3: Single concentration inhibition screening of peptide analogs (10 µM) against M^{pro} and PL^{pro} from SARS-COV-2. …………………...…………………... S4

Figure S4: K_d values determination by microscale thermophoresis (MST). (**B**) Plot of the correlation between the assessed IC_{50} and K_d values of peptide analogs **(pBthTX-I)$_2$K**, **A1**, **A2**, **A4**, **A3**, **A5**, **A6**, **A11**, and **A12** (r^2 = 0.7)………..…………S5

Figure S5: Predicted top 2 (**A**), top 3 (**B**), top 4 (**C**), and top 5 (**D**) binding modes of **(pBthTX-I)$_2$K** to PL^{pro}. The binding energies and r.m.s.d. values (with respect to the top-ranked pose) for each pose are indicated..……………………………………...S6

Figure S6. Peptides' integrity assessment. Analytical HPLC was performed using a binary HPLC system from Shimadzu with a SPD-10AV Shimadzu UV-Vis detector, coupled to an Ultrasphere C-18 column (5 µ, 4.6 x 250 mm) which was eluted with solvent systems A1 (TFA/H2O, 1:1000) and B1 (ACN/ H2O/TFA, 900:100:1) at a flow rate of 1.0 mL/min and a 5-80% gradient of B1 over 10 min. The HPLC column eluates were monitored by their absorbance at 220 nm The molecular weight was checked by mass spectrometry 2020 (Shimadzu)…………………………………... S7–S8

Figure S7: SARS-CoV-2 PLpro purification. **A)** Chromatogram of Size Exclusion Chromatography step; **B)** SDS-PAGE gel of PLpro: M – molecular weight marker; GF – fraction eluted at Size Exclusion Chromatography step…………..…………...... S9

Figure S8. Representative time-dependent traces of the substrate fluorescence at different inhibitor concentrations………….………………………………………......S10

Figure S9. IC_{50} plots of representative peptides collected in November/2020 and June/2021…………………………………………………………………….... S11

Table S1: Inhibitory activity of **(pBthTX-I)$_2$K** and analogs against SARS-CoV-2 and PL^{pro} at 100 µM and 10 µM, respectively. The substituted Ala residue on the sequence of each peptide is indicated as red…………...………………………………S12

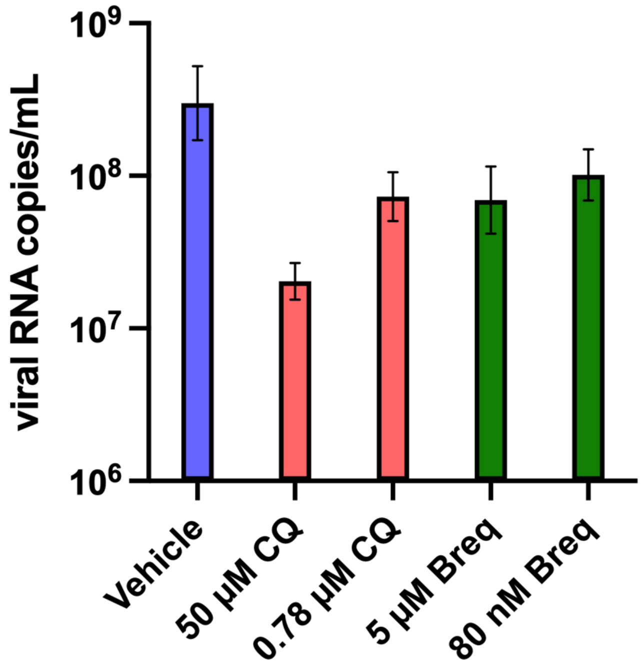

Figure S1. RT-qPCR quantification of antiviral activity of reference drugs against SARS-CoV-2. Viral genome-equivalents in culture supernatants after 48 h of infection in the presence of vehicle (0.5% DMSO, light purple), 50 µM and 0.78 µM chloroquine (salmon) and 5 µM and 80 nM brequinar (green). The mean and standard error of the mean values from three independent experiments are shown.

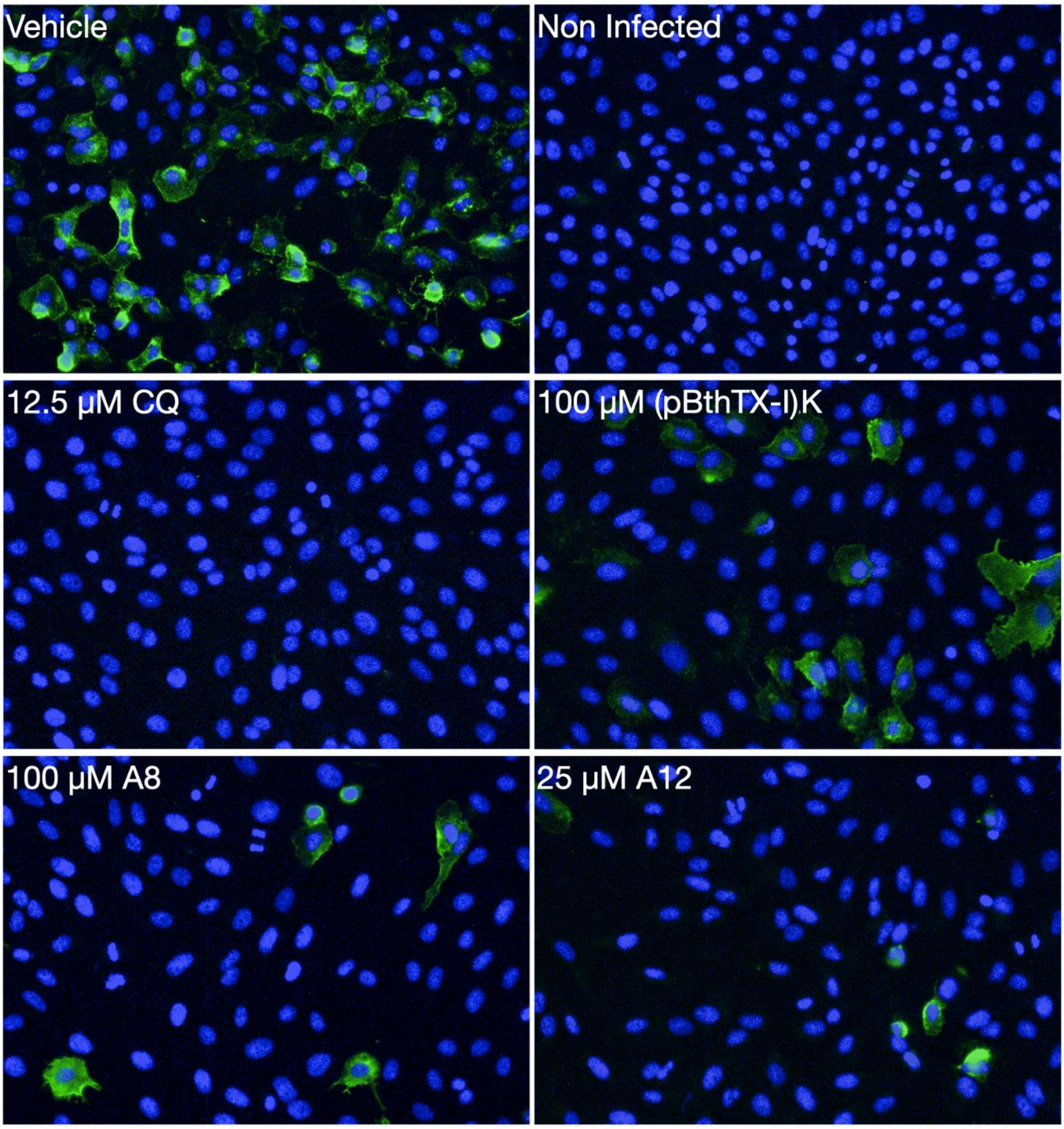

Figure S2. *In vitro* antiviral activity of peptides against SARS-CoV-2-infected Vero cells in the phenotypic screening assay. Representative images of infected cells treated with vehicle (0.5% DMSO, top left), 12.5 µM chloroquine (CQ, middle left), 100 µM $(pBthTX\text{-}I)_2K$ (middle right), 100 µM A8 (bottom left) and 25 µM A12 (bottom right). SARS-CoV-2 infection is shown in green, as detected by indirect immunofluorescence assay (IFA) using an anti-S protein serum. Vero cell nuclei are shown in blue. Non-infected cells are shown as IFA control.

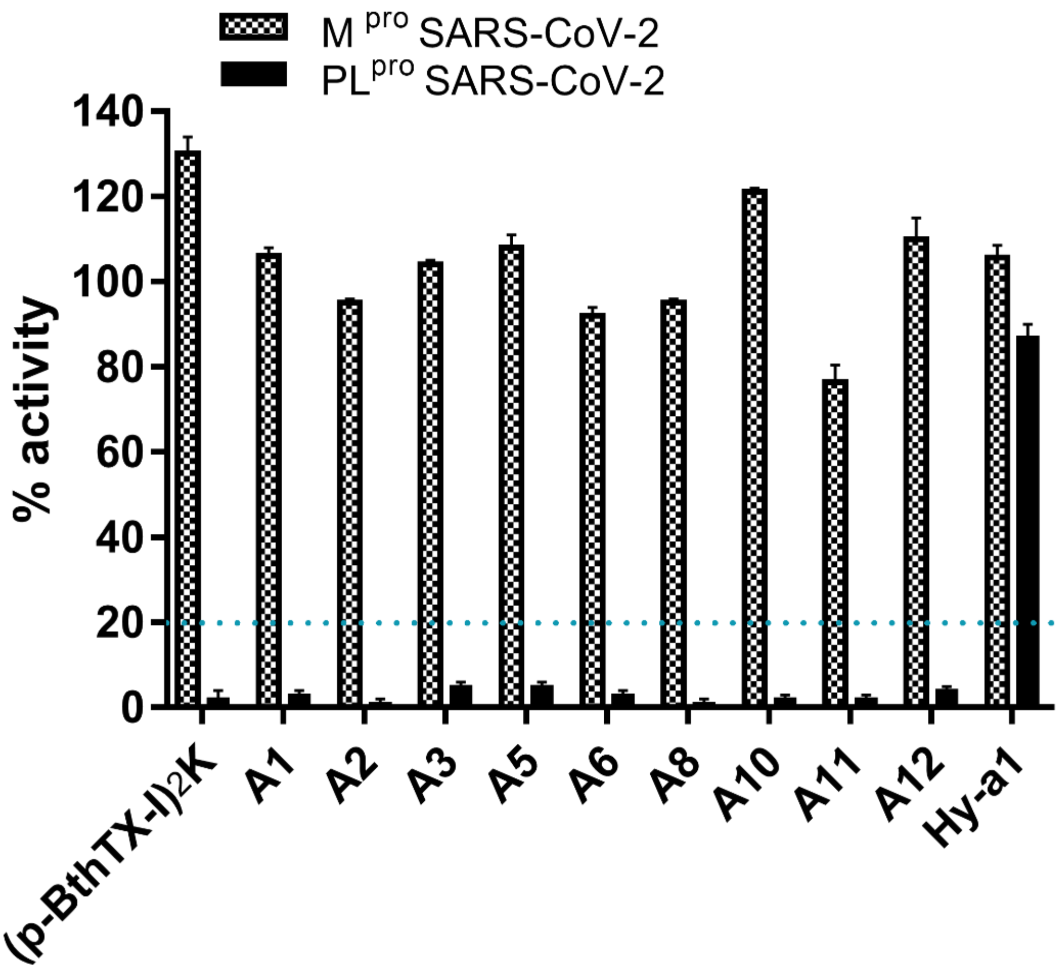

Figure S3: Single concentration inhibition screening of peptide analogs (10 µM) against M^{pro} and PL^{pro} from SARS-COV-2.

A

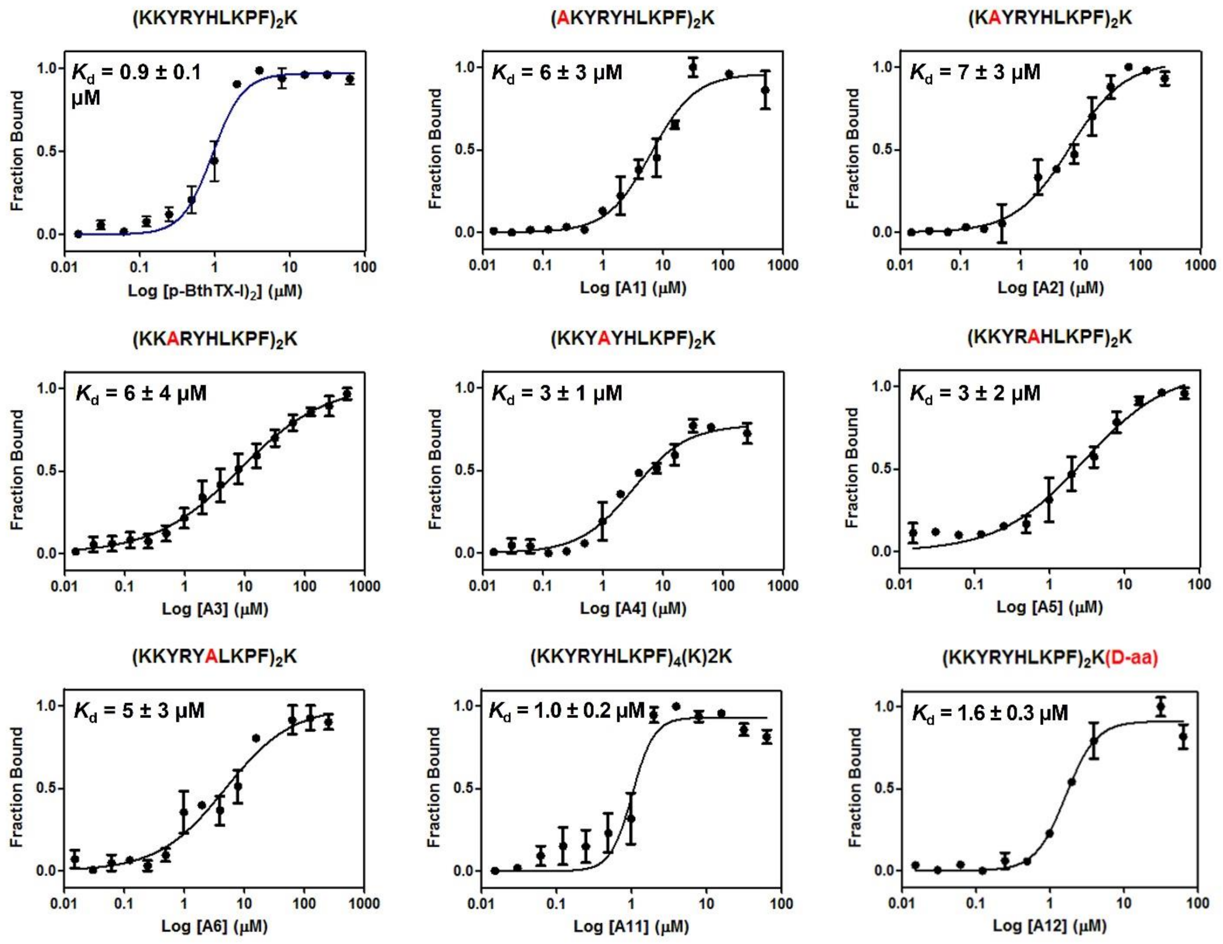

B

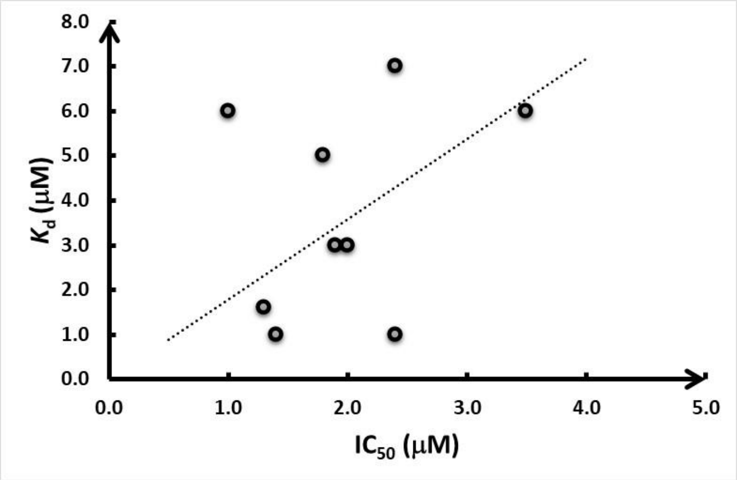

Figure S4: (A) K_d values determination by microscale thermophoresis (MST). (**B**) Plot of the correlation between the assessed IC_{50} and K_d values of peptide analogs **(pBthTX-I)$_2$K**, **A1**, **A2**, **A4**, **A3**, **A5**, **A6**, **A11**, and **A12** (r^2 = 0.7).

A

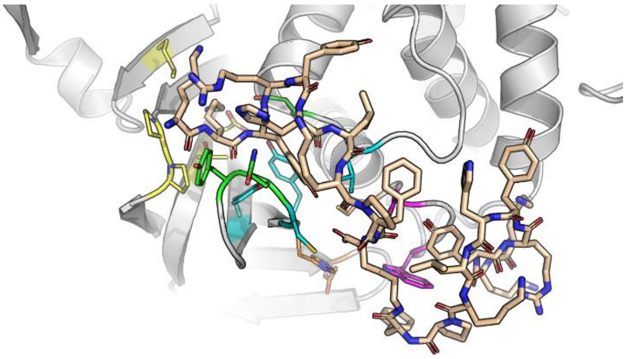

pose 2
Binding energy = -5.1 kcal/mol
r.m.s.d = 2.2 Å

B

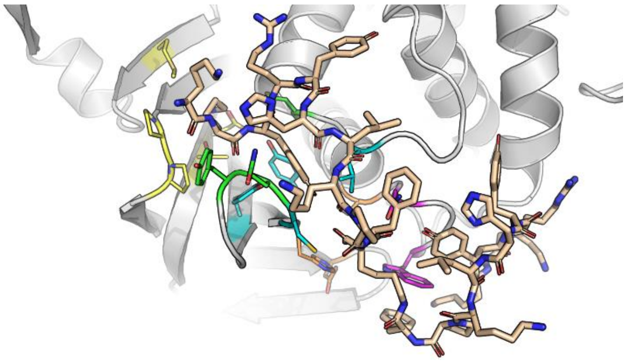

pose 3
Binding energy = -4.8 kcal/mol
r.m.s.d = 2.4 Å

C

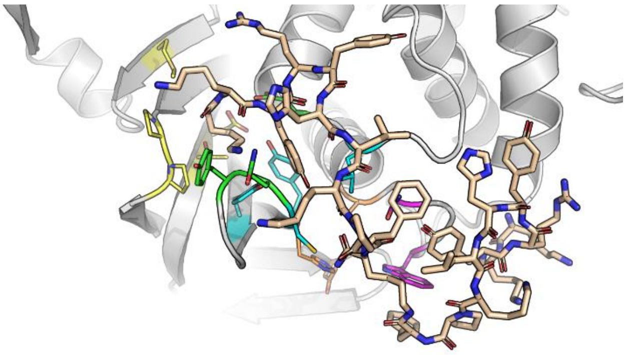

pose 4
Binding energy = -4.8 kcal/mol
r.m.s.d = 2.8 Å

D

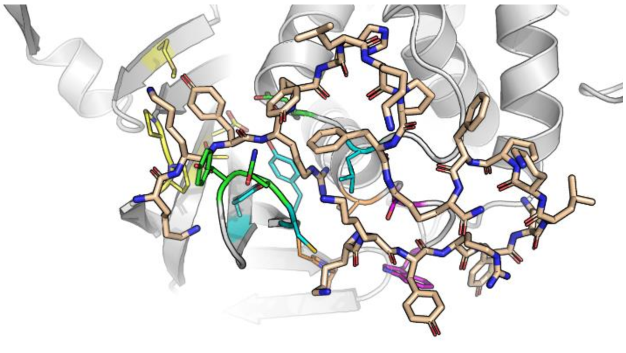

pose 5
Binding energy = -4.7 kcal/mol
r.m.s.d = 3.7 Å

Figure S5: Predicted top 2 (**A**), top 3 (**B**), top 4 (**C**), and top 5 (**D**) binding modes of **(pBthTX-I)$_2$K** to PL^{pro}. The binding energies and r.m.s.d. values (with respect to the top-ranked pose) for each pose are indicated.

(pBthTX-I)2K

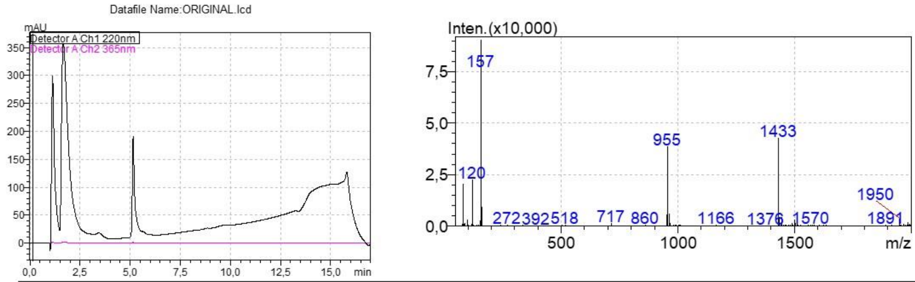

M+ 1H⁺ = 2866
M+2H⁺ = 1433
M+3H⁺ = 955

A2

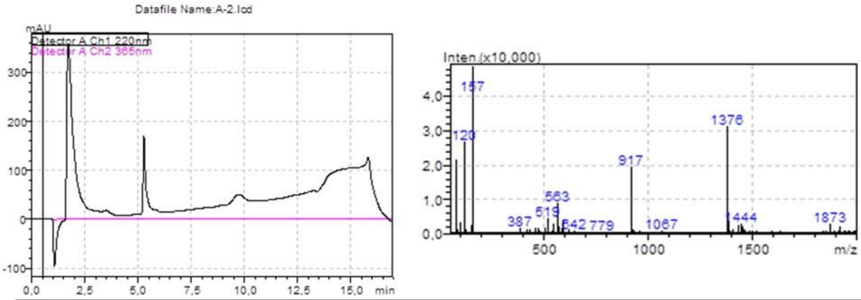

M+ 1H+ = 2752
M+2H+= 1376
M+3H+= 917

A3

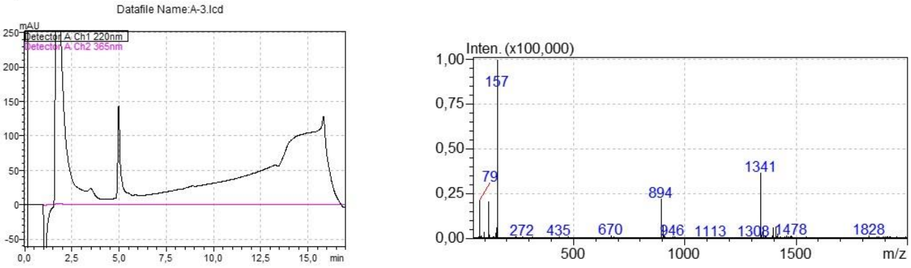

M+ 1H⁺ = 2682
M+2H⁺ = 1341
M+3H⁺ = 894

A4

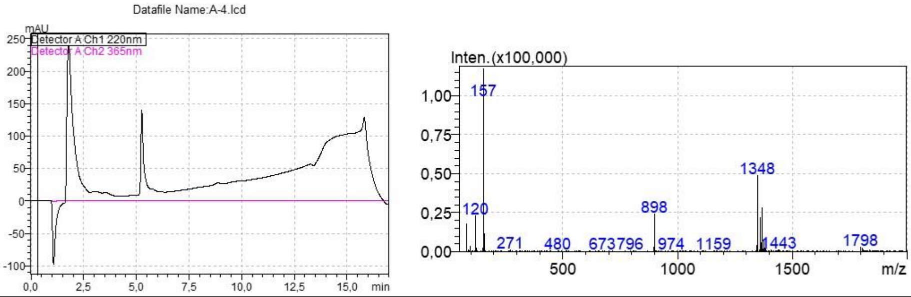

M+ 1H⁺ = 2696
M+2H⁺ = 1348
M+3H⁺ = 898

A5

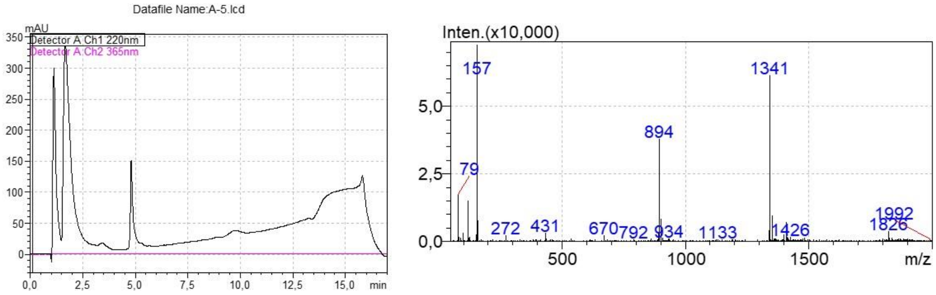

M+ 1H⁺ = 2682
M+2H⁺ = 1341
M+3H⁺ = 894

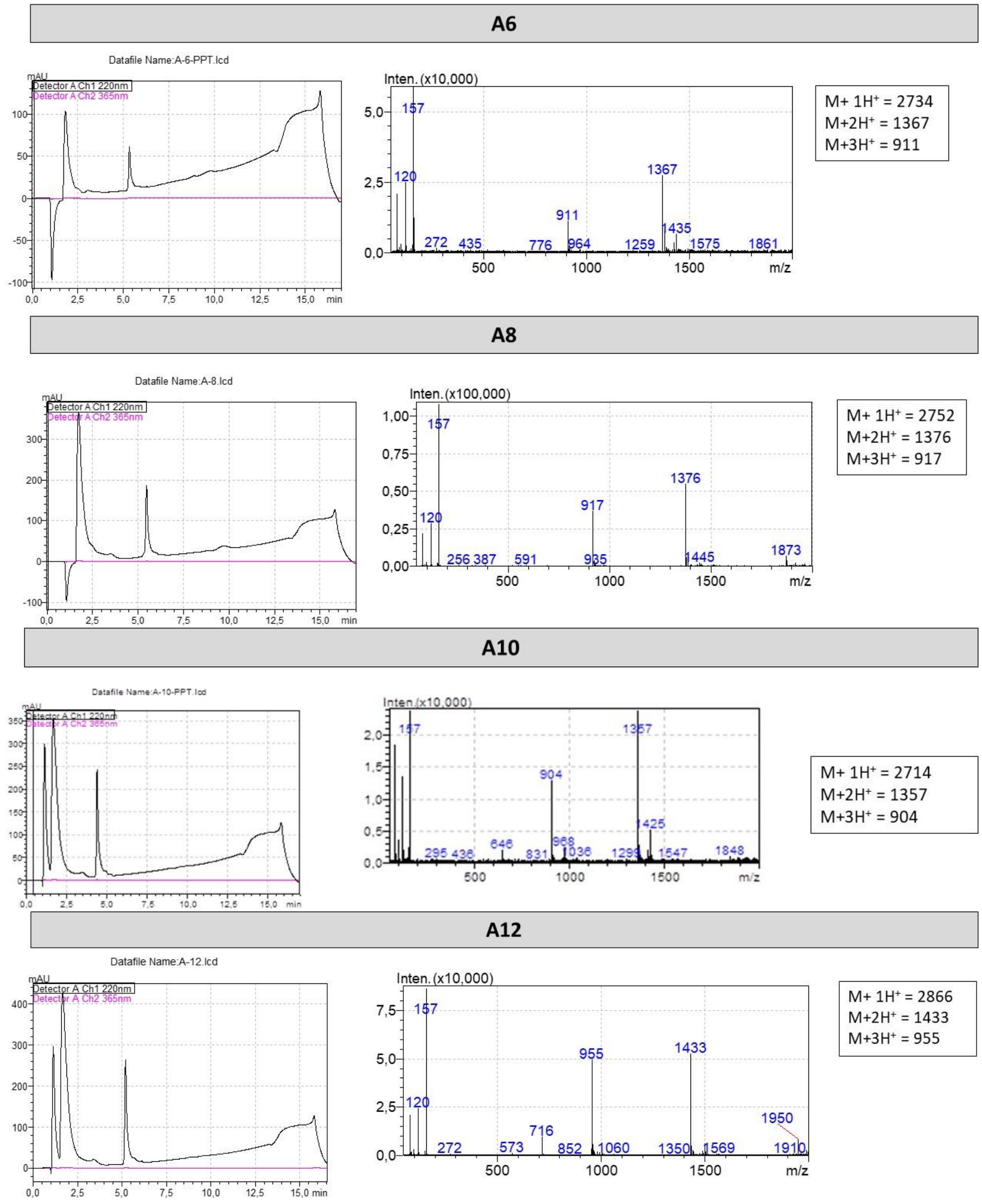

Figure S6. Peptides integrity assessment. Analytical HPLC was performed using a binary HPLC system from Shimadzu with a SPD-10AV Shimadzu UV-Vis detector, coupled to an Ultrasphere C-18 column (5 µ, 4.6 x 250 mm) which was eluted with solvent systems A1 (TFA/H2O, 1:1000) and B1 (ACN/ H2O/TFA, 900:100:1) at a flow rate of 1.0 mL/min and a 5-80% gradient of B1 over 10 min. The HPLC column eluates were monitored by their absorbance at 220 nm The molecular weight was checked by mass spectrometry 2020 (Shimadzu).

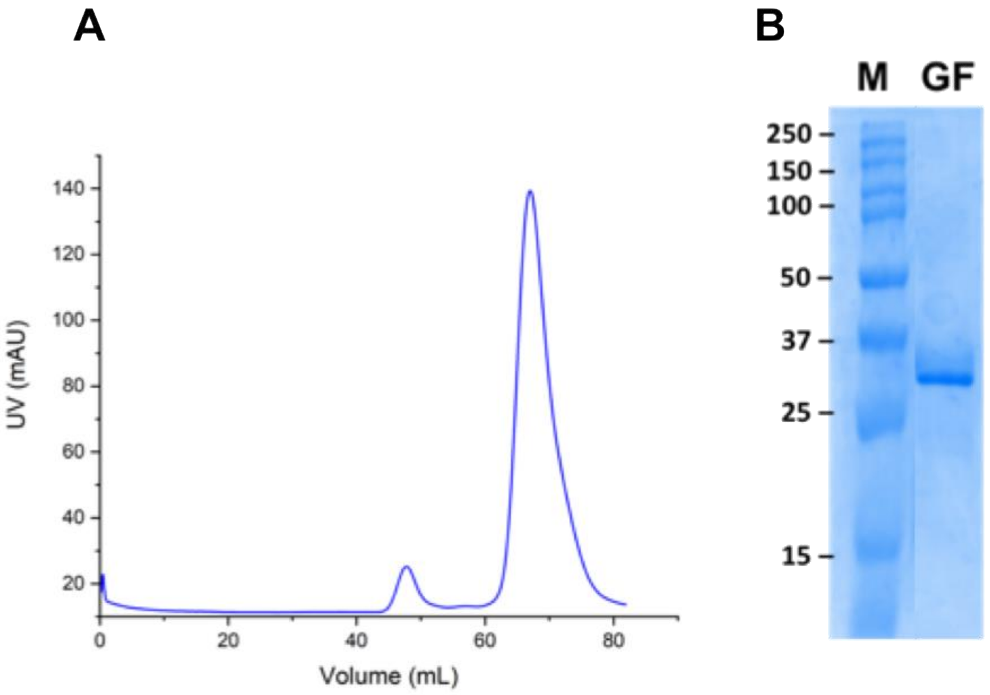

Figure S7: SARS-CoV-2 PL^{pro} purification. **A)** Chromatogram of Size Exclusion Chromatography step; **B)** SDS-PAGE gel of PLpro: M – molecular weight marker; GF – fraction eluted at Size Exclusion Chromatography step.

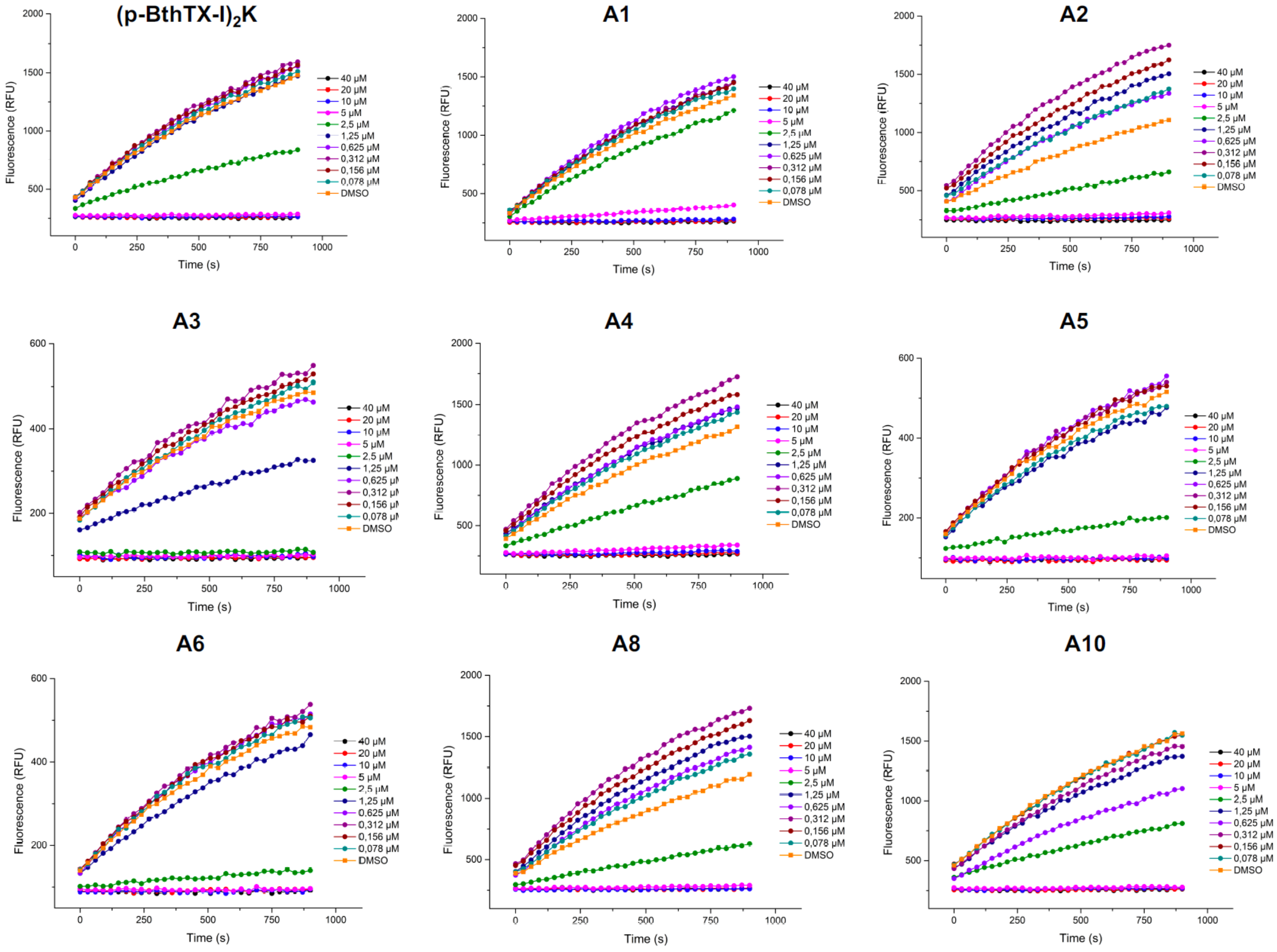

Figure S8. Representative time-dependent traces of the substrate fluorescence at different inhibitor concentrations.

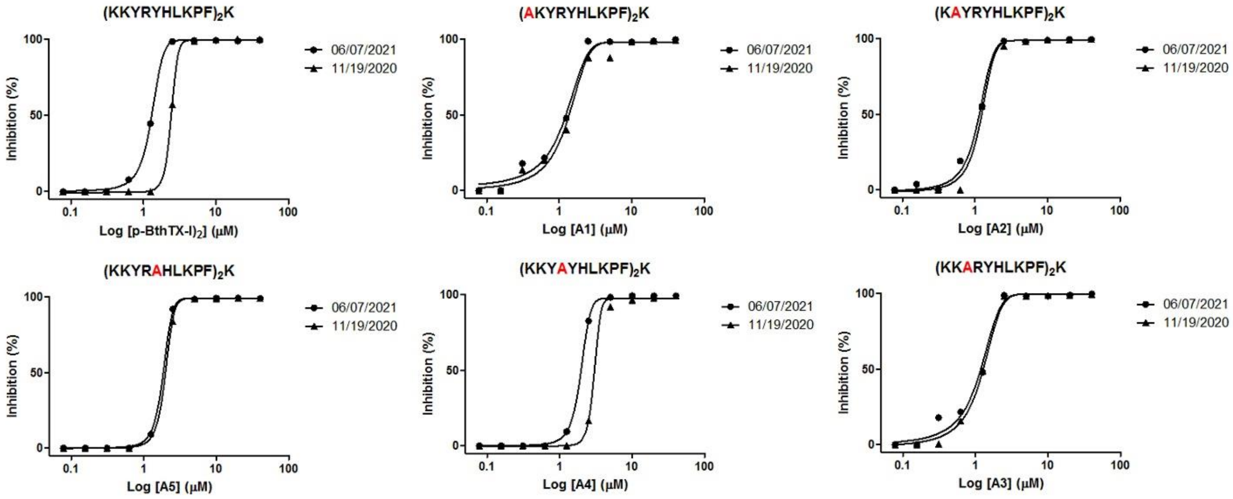

Figure S9. IC_{50} plots of representative peptides collected in November/2020 and June/2021.

Table S1: Inhibitory activity of **(pBthTX-I)$_2$K** and analogs against SARS-CoV-2 and PL^{pro} at 100 µM and 10 µM, respectively. The substituted Ala residue on the sequence of each peptide is indicated as red.

Code	Peptide Sequence	M.W. (Da)	SARS-CoV-2 % of inhibition at 100 µM	PL^{Pro} % of inhibition at 10 µM
**(pBthTX-I)$_2$K**	(KKYRYHLKPF)$_2$K	2868	27	98 ± 2
**A1**	(**A**KYRYHLKPF)$_2$K	2754	4	97 ± 1
**A2**	(K**A**YRYHLKPF)$_2$K	2754	10	99 ± 1
**A3**	(KK**A**RYHLKPF)$_2$K	2684	57	95 ± 1
**A4**	(KKY**A**YHLKPF)$_2$K	2698	10	97 ± 1
**A5**	(KKYR**A**HLKPF)$_2$K	2684	70	95 ± 1
**A6**	(KKYRY**A**LKPF)$_2$K	2736	22	97 ± 1
**A8**	(KKYRYHL**A**PF)$_2$K	2754	29	99 ± 1
**A10**	(KKYRYHLKP**A**)$_2$K	2716	7	98 ± 1
**A11**	(KKYRYHLKPF)$_4$(K)2K	5848	54	98 ± 1
**A12**	(KKYRYHLKPF)$_2$K(d-aa)	2868	69	96 ± 1
**Hy-a1**	IFGAILPLALGALKNLIK-NH$_2$	1865	59	13 ± 3

n.d. = not determined
